# Efficacy of switching from teriparatide to zoledronic acid or denosumab on bone mineral density and biochemical markers of bone turnover in older patients with severe osteoporosis: a real-life study

**DOI:** 10.1007/s12020-023-03431-6

**Published:** 2023-07-04

**Authors:** Giorgia Dito, Marina Lugaresi, Chiara Degradi, Gregorio Guabello, Matteo Longhi, Sabrina Corbetta

**Affiliations:** 1 Endocrinology and Diabetology Service, IRCCS Istituto Ortopedico Galeazzi, Milan, Italy; 2 Department of Biotechnology and Translational Medicine, University of Milan, Milan, Italy; 3 Department of Medical and Surgical Pathophysiology and Transplantation, University of Milan, Milan, Italy; 4 Rheumatology Unit, IRCCS Istituto Ortopedico Galeazzi, Milan, Italy; 5 Bone Metabolism Diseases and Diabetes Unit, IRCCS Istituto Auxologico Italiano, Milan, Italy; 6 Department of Biomedical, Surgical and Dental Sciences, University of Milan, Milan, Italy

**Keywords:** Bone mineral density, Denosumab, Fragility fractures, Osteoporosis, Teriparatide, Zoledronic acid, Severe osteoporosis

## Abstract

**Purpose:**

Osteoporosis is characterized by loss of bone mass and susceptibility to fracture. Skeletal effects of teriparatide (TPT) are not persistent after drug withdrawal and sequential therapy with bisphosphonates or denosumab (Dmab) after TPT discontinuation represents a valid option. Here, the two sequential strategies were evaluated in severe osteoporotic patients.

**Methods:**

The study retrospectively enrolled 56 severe osteoporotic patients who received TPT for 24 months followed by 24 months of zoledronic acid (ZOL) (TPT + ZOL) or Dmab (TPT+Dmab). Clinical features, incident fractures, bone mineral density (BMD) measurements, and bone marker profiles were collected. One-way ANOVA analyzed the difference between mean T-scores at baseline, after 24 months of TPT, and after 2 doses of ZOL or after at least 3 doses of Dmab.

**Results:**

Twenty-three patients received TPT + ZOL (19 females, 4 males; median [IR] age, 74.3 [66.9, 78.6] years) and 33 patients received TPT+Dmab (31 females, 2 males; mean [IR] age, 66.6 ± 11.3 years). Mean lumbar and hip T-scores were increased after both TPT + ZOL and TPT+Dmab (all *p* < 0.05 vs baseline). The size effects induced by TPT + ZOL on the lumbar and hip BMD T-scores were similar to those observed with TPT+Dmab with mean T-scores increases of about 1 and 0.4 SD, respectively. No significant between-group differences were identified. Incident fragility fractures occurred in 3 (13%) patients treated with TPT + ZOL and in 5 (15%) patients treated with TPT+Dmab.

**Conclusions:**

Sequential TPT + ZOL therapy is likely to increase bone mineralization at the lumbar level and to stabilize it at the femoral level, similarly to what obtained with the sequential TPT+Dmab. Both ZOL and Dmab are suggested to be effective sequential treatments after TPT.

## Introduction

Osteoporosis is a chronic condition characterized by a loss of bone mass and increased bone fragility and susceptibility to fracture, affecting virtually all skeletal sites (reviewed in Compston et al., 2019 [1]). As patients often require long-term treatment to maintain optimal bone strength, sequential treatment regimens may represent a valid option to increase bone mineral density (BMD) initially and then maintain it.

Teriparatide (TPT) is a parathyroid hormone analog that stimulates the formation of bone by acting on osteoblasts and increasing calcium absorption [2]. Treatment with TPT reduces the risk of vertebral and non-vertebral fractures and increases vertebral, femoral, and total-body BMD [3]. However, TPT can only be used for up to 24 months, and patients can only receive one course of TPT during their lifetime [2]. Withdrawal of TPT treatment causes the loss of BMD, which can be prevented by sequential treatment with antiresorptive agents [4].

Antiresorptive drugs form the foundation of osteoporosis treatment, and both bisphosphonates (BPs) and denosumab (Dmab), a monoclonal antibody, significantly reduce fracture risk in postmenopausal women [5]. Furthermore, gains in BMD achieved with the anabolic agents, TPT and abaloparatide, are maintained or further increased at all sites when sequential treatment with BPs or Dmab follows; however, the effects on BMD appear to be greater with Dmab than oral BPs [5, 6].

Zoledronic acid (ZOL), also known as zoledronate, is a BPs that is administered intravenously once yearly. The effects of sequential treatment with TPT followed by ZOL, and ZOL followed by TPT, were investigated in ovariectomized rats by Shimizu and colleagues, who reported that switching from TPT to ZOL maintained or further increased BMD [7]. To date, two studies have assessed sequential treatment with TPT followed by ZOL in postmenopausal women [8, 9]. Kocjan et al. reported significantly greater BMD gains at the lumbar spine with Dmab compared with BPs sequential to TPT in postmenopausal women with severe primary osteoporosis; however, only 31% (22 of 70) of patients treated with BPs received ZOL [8]. Conversely, bone turnover markers and BMD were affected to a similar degree with sequential treatment with Dmab (at least two doses) or ZOL (single 5 mg dose given intravenously) in 28 osteoporotic postmenopausal women with a very high risk of fractures who had completed at least 12 months of treatment with TPT [9]. Hence, the optimal sequential treatment strategy after TPT is undetermined.

The present study aimed to evaluate the effect of sequential treatment with TPT followed by ZOL or TPT followed by Dmab on the lumbar, femur neck, and hip BMD and on circulating bone and mineral markers in patients with osteoporosis and severe fragility fractures. At variance with previous studies, a complete 24 months course of TPT treatment was administered to each participants followed by at least 24 months of ZOL or Dmab therapy. Moreover, enrolled patients were all affected with severe osteoporosis, having experienced at least one fragility fracture, and at very high risk of re-fracture.

## Materials and methods

### Study design

This retrospective study included severe osteoporotic patients with fragility fractures who were referred for osteoporosis management to the third-level Endocrinology Service and Rheumatology Unit at the IRCCS Galeazzi Orthopedic Institute in Milan, Italy between 2012 and 2018. Clinical data were derived from the OsteoRegistry database, founded by the 5 × 1000 GSD Foundation. Criteria of inclusion were:

1. treatment with TPT 20 μg for 24 months with a compliance higher than 80%
2. treatment with at least 2 doses of ZOL 5 mg iv
3. treatment with at least 3 doses of Dmab 60 mg sc
4. availability of DEXA measurement by Hologic or Lunar devices at baseline, at the end of TPT treatment and after 24 months of ZOL or Dmab therapy.

All patients had experienced at least one fragility fracture and were treated with TPT according to the criteria of the Italian nota AIFA 79, which, for reimbursement, includes patients with: (1) three fragility fractures; (2) T-scores less than −4.0 plus one fragility fracture; (3) one fragility fracture on at least 12 months-treatment with BPs; or (4) at least one fragility fracture on 12 months-treatment with prednisone ≥5 mg/day or equivalent.

Patients with active or previous neoplasia, metastatic disease, estimated glomerular filtration rate (eGFR) < 30 ml/min, heart failure in NYHA classes 3–4, cirrhosis, or who had received prior treatment with aromatase inhibitors were excluded.

**Fig. 1:**
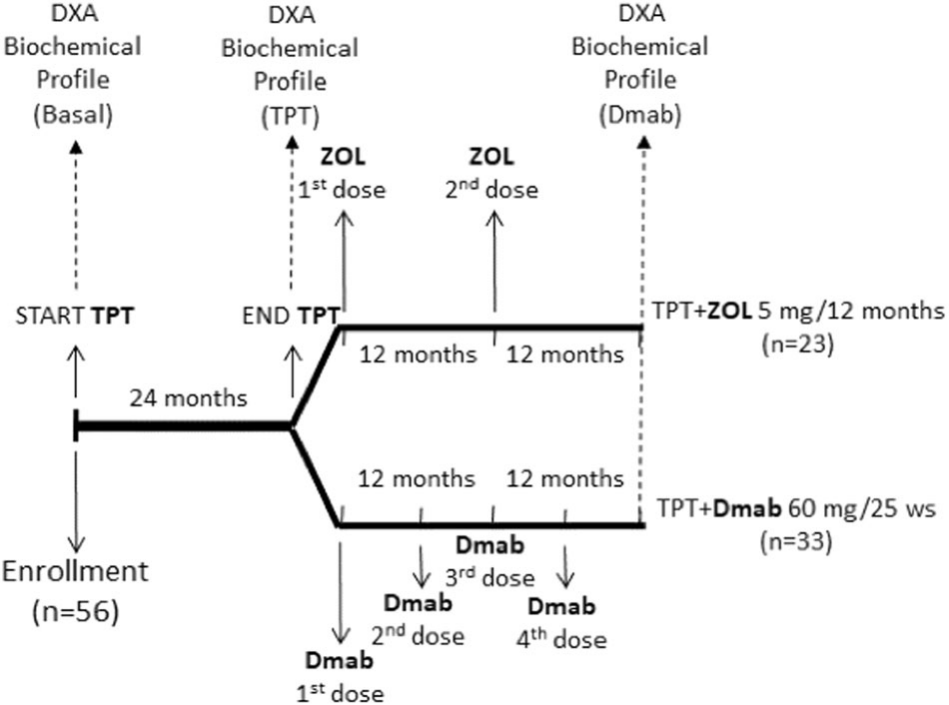
Study design. DXA, dual-energy x-ray absorptiometry at lumbar, femur neck, and hip sites; TPT teriparatide 20 μg/day subcutaneously; ZOL zoledronic acid 5 mg/12 months intravenously; Dmab denosumab 60 mg/25 weeks subcutaneously

All patients received 24 months of TPT 20 μg subcutaneously once daily followed by sequential treatment with either ZOL 5 mg given intravenously every 12 months for 24 months (two doses) (TPT + ZOL group) or Dmab 60 mg administered as a single subcutaneous injection every 25 weeks for 24 months (four doses) (TPT+Dmab group). Patients were evaluated at baseline, after 24 months of treatment with TPT, and after 24 months of treatment with either ZOL 5 mg or Dmab 60 mg (Fig. 1). Patients were assigned to ZOL or Dmab therapy by the referent clinician; indeed, clinicians were more prone to treat patients with severe demineralization with Dmab.

Demographic characteristics, medical history, and clinical data, including age and weight, were collected at baseline. Patients were also evaluated for the presence of morphometric vertebral fractures by routine dorsal and lumbar x-ray imaging at baseline.

Biochemical parameters, including serum total calcium, serum phosphate, plasma parathormone (PTH), serum total alkaline phosphatase (ALP), serum carboxy-terminal collagen crosslinks (βCTX), and serum 25 hydroxyvitamin D (25OHD), were collected at baseline, after 24 months of TPT treatment, and after 24 months of treatment with either ZOL or Dmab. Serum 25OHD levels were measured at each clinical assessment and corrected by oral supplementation with cholecalciferol or calcifediol to maintain serum 25OHD levels above the threshold of 30 ng/ml.

Circulating bone and mineral markers were measured by commercially available assay kits in outpatient diagnostic sources.

BMD measurements of the lumbar, femur neck, and hip were performed using dual-energy x-ray absorptiometry (DXA; Hologic or Lunar GE systems) at baseline, after 24 months of TPT treatment, and after 24 months of treatment with either ZOL or Dmab. Each enrolled patient was repeatedly measured by the same system (Hologic or Lunar GE). BMD measurements were expressed as T-scores. T-score was calculated by third National Health and Nutrition Examination Survey, using data of young white female as reference [10]. For all lumbar T-scores, vertebral bodies not affected by osteoporotic deformations or arthrosis/arthritis were considered and lumbar T-scores excluding more than 2 vertebral bodies from the evaluation due to artifacts were not included in the analysis.

Occurrence of clinical fragility fractures or morphometric vertebral fractures during the 48-month study period were recorded.

### Statistical analysis

The primary aim of the study was to test the hypothesis that the changes (Δ) in lumbar T-scores after the two different sequential treatments were similar. The sample size was calculated after data collection: an actual power (1-β error probability) of 0.81, considering an α-error probability of 0.05 and an effect size of 0.6 by G*Power3.1 characterized the analysis of the difference between the Δ in lumbar T-scores at the end of the sequential treatments.

Demographic, clinical, and biochemical parameters passing the normality test are presented as mean ± standard deviation (SD), while non-parametric variables are presented as median (interquartile range [IR]).

Ordinary one-way ANOVA adjusted for multiple testing was performed to analyze the differences between the median T-score at three evaluation time points when variables failed the normality test. For the TPT + ZOL group, evaluations were at baseline, after 24 months of TPT, and after 2 doses of ZOL. For the TPT+Dmab group, evaluations were at baseline, after 24 months of TPT, and after at least 3 doses of Dmab. A correlation between two variables was analyzed by the Pearson or Spearman test, as appropriate. Data were analyzed by using Past 3.14 and Prism 6.0. A *p*-value of <0.05 was considered statistically significant.

## Results

### Patient characteristics

This study enrolled 56 severe osteoporotic patients with fragility fractures (50 post-menopausal women and 6 men). After completing 24 months of treatment with TPT, 23 patients continued treatment with ZOL (TPT + ZOL group), and 33 patients continued treatment with Dmab (TPT+Dmab group).

The baseline characteristics of both patient groups are shown in Table 1. The TPT + ZOL group included 19 females and 4 males, with a median (IR) age of 74.3 (66.9, 78.6) years overall and 50.0 (47.0, 51.5) years at menopause. The TPT+Dmab group included 31 females and 2 males [median (IR) age, 65.0 (59.5, 77.5) years; median (IR) age at menopause, 48.0 (42.0, 50.0) years]. Approximately one-third (35%) of TPT + ZOL patients and one-quarter (24%) of TPT+Dmab patients were active or previous smokers.

**Table 1:** Baseline clinical and biochemical characteristics of patients treated with sequential TPT + ZOL or TPT+Dmab

Parameters	n.v.	TPT + ZOL	TPT+Dmab	*p*-value
Number of patients		23	33	
Female/Male, *n*		19/4	31/2	0.215
Age, years (range)		74.3 (66.9, 78.6)	65.0 (59.5, 77.5)	0.134
Age at menopause, years (range)		50.0 (47.0, 51.5)	48.0 (42.0, 50.0)	0.217
Weight, kg (mean ± SD)		61.0 ± 11.3	61.3 ± 10.7	0.911
Active smokers		3 (13.0)	3 (9.1)	0.681
Ex-smokers		5 (21.7)	5 (15.1)	0.725
Comorbidities and previous treatments, *n* (%)				
Diabetes		2 (8.7)	0 (0.0)	0.164
RA/undifferentiated connectivities		1 (4.3)	1 (3.0)	1.000
COPD		2 (8.7)	1 (3.0)	0.562
IBD		1 (4.3)	0 (0.0)	0.411
Steroids		3 (13.0)	1 (3.0)	0.295
Previous steroids		4 (17.4)	4 (12.1)	0.704
Previous anti-OP treatment		13 (56.5)	23 (69.7)	0.398
Bone features, *n* (%)				
Femur fractures (≥1)		4 (17.4)	3 (9.1)	0.429
Vertebral fractures (≥1)		23 (100)	33 (100)	1.000
Vertebral fractures (total *n*)		3 (2, 5)	4 (3, 5)	0.289
Non-femur non-vertebral Fx (≥1)		4 (17.4)	6 (18.2)	1.000
Circulating bone markers				
Serum total Ca (mg/dl)	8.4–10.4	9.4 ± 0.4	9.4 ± 0.4	0.909
Serum P (mg/dl)	3.0–5.0	3.3 ± 0.5	3.4 ± 0.6	0.844
Plasma PTH (pg/ml)	15–65	48.4 ± 13.9	46.7 ± 16.4	0.689
Serum total ALP (U/L)	30–120	71.0 (64.0, 90.0)	78.5 ± 23.4	0.707
Serum βCTX (ng/ml)		0.308 ± 0.274	0.336 (0.175, 0.475)	0.147
Serum 25OHD (ng/ml)	30–50	26.0 (19.3, 34.3)	31.5 (26.0, 39.8)	0.083
Bone mineral density				
Lumbar T-score (SD)		−3.06 ± 1.15	−3.82 ± 0.80	**0.028**
Femur neck T-score (SD)		−2.66 ± 1.00	−2.54 ± 0.75	0.616
Hip T-score (SD)		−2.26 ± 1.16	−2.36 ± 0.97	0.755

Data are presented as *n* (%), mean ± SD for normally distributed data, or median (interquartile range) for data that failed the normality test.

*ALP* alkaline phosphatase, *Ca* calcium, *COPD* chronic obstructive pulmonary disease, *βCTX* carboxy-terminal collagen crosslinks, *Dmab* denosumab, *IBD* inflammatory bowel disease, *n* number, *n.v.* normal values, *OP* osteoporosis, *P* phosphate, *PTH* parathormone, *RA* rheumatoid arthritis, *SD* standard deviations, *TPT* teriparatide, *ZOL* zoledronate, *25OHD* 25 hydroxyvitamin D, *Fx* fractures.

In the TPT + ZOL group, 30.4% (*n* = 7) of patients had received long-term (i.e., more than 6 months) steroid treatment, which was ongoing in 3 patients at enrollment. In the TPT+Dmab group, 15.1% (*n* = 5) of patients had received long-term steroid treatment, with 1 patient still receiving steroids at enrollment.

All patients had experienced at least one vertebral fracture. The median (IR) number of vertebral fractures in the TPT + ZOL group was 3 (2, 5), and 4 (3, 5) in the TPT+Dmab group. Femur fractures were reported by 4 patients (17.4%) in the TPT + ZOL group and 3 patients (9.1%) in the TPT+Dmab group; 17.4% and 18.2% of patients, respectively, reported at least one non-femur non-vertebral fracture. Previous anti-osteoporotic treatment was reported by 56.5% and 69.7% of patients in the TPT + ZOL and TPT+Dmab groups, respectively.

There were no significant between-group differences in patient age, age at menopause, gender, weight, co-morbidities, steroid use, smoking, or prior treatment for osteoporosis. Conversely, patients in the TPT+Dmab group had significantly lower mean lumbar T-scores at baseline than those in the TPT + ZOL group (*p* = 0.028).

### Changes in BMD and new bone fractures during sequential treatment

**Fig. 2:**
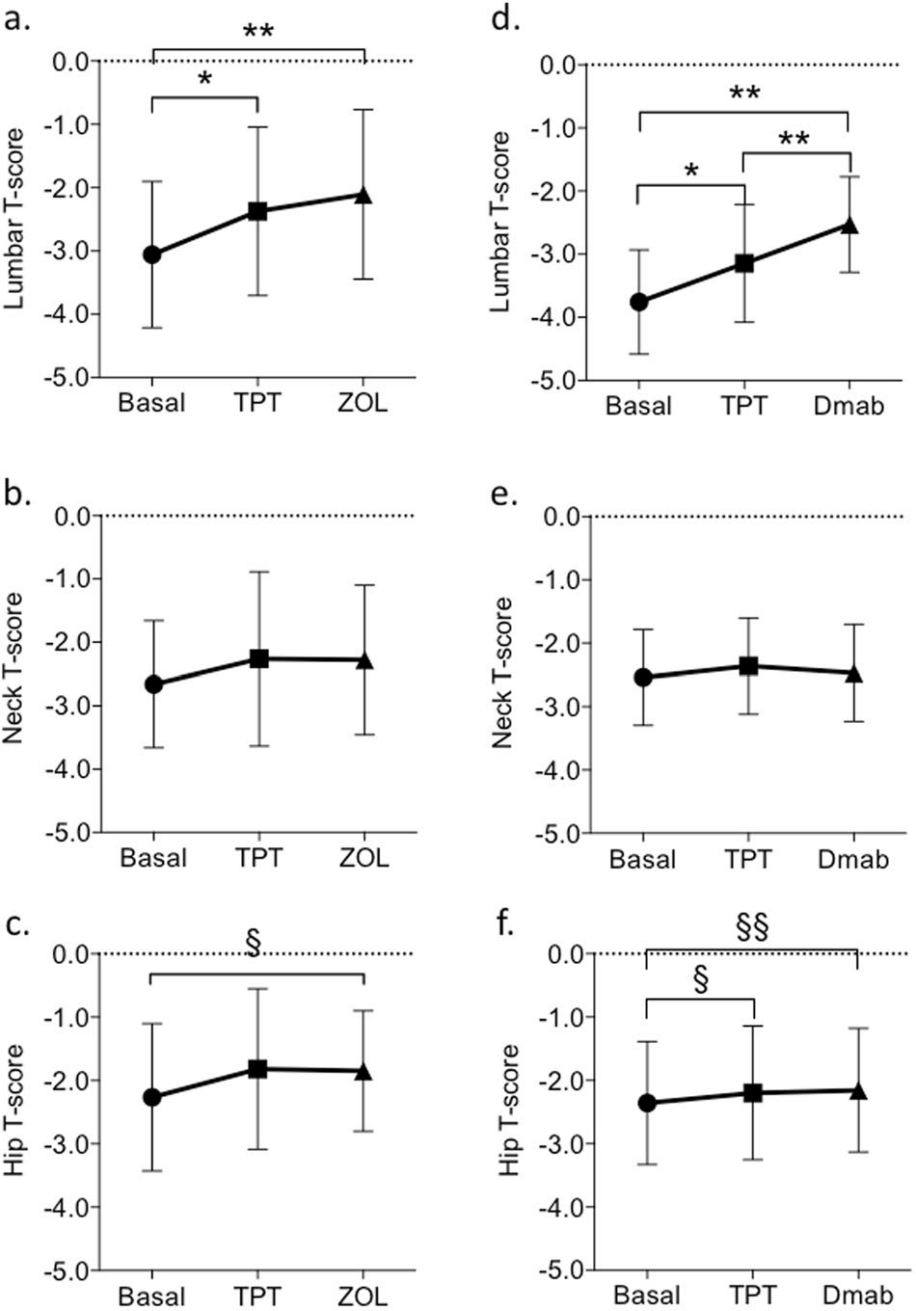
Effect of sequential treatment with TPT + ZOL versus TPT+Dmab on lumbar, femur neck, and hip BMD T-scores. Sequential treatment with TPT + ZOL determined significant increases in mean lumbar (**a**) and hip (**c**) T-scores, while no significant change was detected in the mean neck T-score (**b**). Similarly, sequential treatment with TPT+Dmab determined significant increases in mean lumbar (**d**) and hip (**f**) T-scores, while no significant change was detected in the mean neck T-score (**e**). Panel (**a**), *p < 0.0001; **p = 0.0007; Panel (**c**), ^§^*p* = 0.0492; Panel (**d**), *p < 0.0001; **p = 0.0074; Panel (**f**), ^§^*p* < 0.0001; ^§§^*p* < 0.0001; TPT, 20 μg/day sc teriparatide; ZOL, 5 mg/12 months iv zoledronic acid; Dmab, 60 mg/25 weeks sc denosumab

During TPT treatment all patients were clinically re-evaluated by outpatient visits and bone markers were checked every 6 months, as required by Italian agency AIFA for TPT reimbursement. Compliance with TPT treatment, evaluated on patients’ declarations, was high, with >90% of doses administered. After 24 months of treatment with TPT, the mean ± SD lumbar T-score increased by 22 ± 27% in TPT + ZOL patients and by 17 ± 18% in TPT+Dmab patients compared with baseline. Similarly, the mean ± SD femoral neck and hip T-scores increased by 2 ± 21% and 14 ± 19%, respectively, in the TPT + ZOL group and by 5 ± 22% and 13 ± 43%, respectively, in the TPT+Dmab group (Fig. 2). There were no significant between-group differences in the mean gain in T-scores from baseline to 24 months at any site.

Sequential ZOL treatment after TPT showed a trend to increase the lumbar T-score from a mean ± SD T-score of −2.37 ± 1.33 after TPT to −2.11 ± 1.34 after ZOL treatment (Fig. 2a, *p* = 0.181). Indeed, increases in lumbar T-score could be detected in 53% of patients (from 10 to 129%), while stabilization of lumbar T-score was observed in 16% of patients. Mean ± SD femur neck T-scores did not change after treatment with ZOL (−2.26 ± 1.38 vs −2.28 ± 1.18; Fig. 2b), though increases in neck T-score could be detected in 55% of patients (from 3 to 164%), while stabilization of neck T-score was observed in 18% of patients. Similarly, mean ± SD hip T-scores did not change after treatment with ZOL (−1.82 ± 1.27 vs −1.85 ± 0.95; Fig. 2c), while increases in hip T-score could be detected in 68% of patients (from 3 to 84%), and stabilization of hip T-score was observed in 9% of patients.

With respect to baseline T-scores, the mean lumbar T-score was significantly higher after sequential TPT + ZOL treatment (*p* = 0.0007) with a Δ T-score of 0.92 ± 0.75, as was the mean hip T-score (*p* = 0.0492) with a Δ T-score of 0.41 ± 0.82.

Sequential Dmab treatment after TPT increased the mean ± SD lumbar T-score from −3.14 ± 0.93 to −2.53 ± 0.76 (Fig. 2d, *p* < 0.0001). Increases in lumbar T-score with respect to T-score gained after TPT treatment, could be detected in 90% of patients (from 6 to 42%). Dmab treatment did not induce changes in the mean ± SD femur neck T-score (from −2.36 ± 0.76 to −2.47 ± 0.76) (Fig. 2e). However, increases in neck T-score could be detected in 73% of patients (from 6 to 42%). Dmab treatment did not induce changes in the mean ± SD hip T-scores (from −2.20 ± 1.06 to 2.15 ± 0.98) (Fig. 2f), but increases in hip T-score could be detected in 84% of patients (from 3 to 57%).

Referring to baseline T-scores, the mean lumbar T-score was significantly higher after sequential TPT+Dmab treatment (*p* = 0.0074) with a Δ T-score of 1.16 ± 0.58, as was the mean hip T-score (*p* < 0.0001) with a Δ T-score of 0.43 ± 0.34. Indeed, looking for clinical conditions predictive of the Δ T-score outcomes, any significant correlation could be detected.

**Fig. 3:**
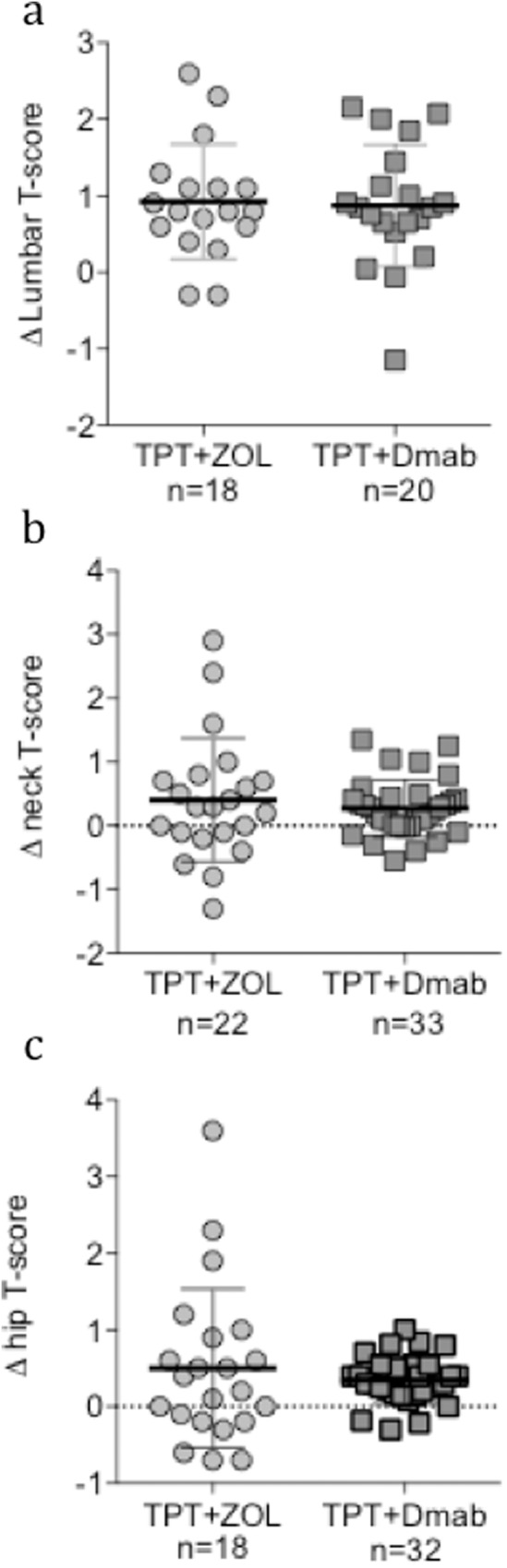
Comparison of sequential treatment-induced BMD T-score changes in lumbar, femur neck, and hip T-scores between TPT-ZOL and TPT+Dmab patients. The change in mean T-score from baseline to the end of sequential treatment was not different between TPT + ZOL and TPT+Dmab groups at any sites. Δ, change in mean T-score; TPT, 20 μg/day sc teriparatide; ZOL, 5 mg/12 months iv zoledronic acid; Dmab, 60 mg/25 weeks sc denosumab; n, number of patients included in the analysis

There were no significant between-group differences in the change in mean lumbar, femur neck, and hip T-scores from baseline to the end of sequential treatment (Fig. 3).

**Fig. 4:**
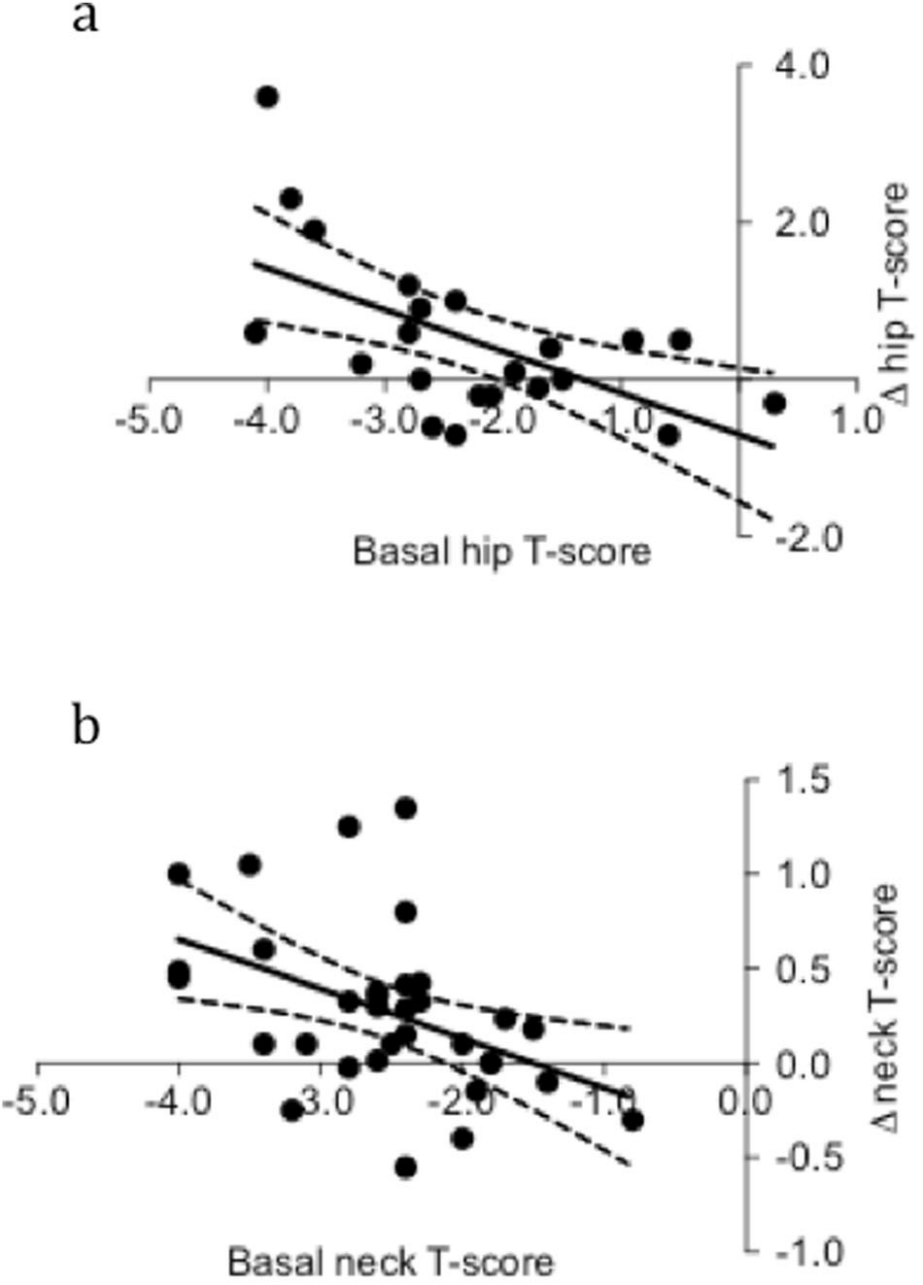
Correlation between baseline T-scores and sequential treatment-induced changes in T-scores. The baseline hip T-score was negatively correlated (r^2^ = 0.351, *p* = 0.004) with the change in hip T-scores after TPT + ZOL sequential treatment (**a**). The baseline femur neck T-score was negatively correlated (r^2^ = 0.200, *p* = 0.009) with the change in neck T-scores after TPT+Dmab sequential treatment (**b**). TPT, 20 μg/day sc teriparatide; ZOL, 5 mg/12 months iv zoledronic acid; Dmab, 60 mg/25 weeks sc denosumab

TPT + ZOL and TPT+Dmab sequential treatments were more effective in increasing BMD T-scores at the hip and femur neck levels, respectively, in patients with very low hip and neck T-scores at enrollment than in those with high hip and neck T-scores (Fig. 4a, b). The baseline hip T-score was negatively correlated (r^2^ = 0.351, *p* = 0.004) with the change in hip T-score after TPT + ZOL sequential treatment (Fig. 4a), while the baseline femur neck T-score was negatively correlated (r^2^ = 0.200, *p* = 0.009) with the change in femur neck T-score after TPT+Dmab sequential treatment (Fig. 4b).

Fragility fractures occurred in 3 (13%) patients treated with TPT + ZOL; 1 patient experienced a femur neck fracture during TPT treatment, while 1 patient experienced a femur neck fracture and another patient experienced a non-femoral and non-vertebral fracture during ZOL treatment. Similarly, fragility fractures occurred in 5 (15%) patients in the TPT+Dmab group; 4 patients reported a vertebral fracture during TPT treatment, while 1 patient reported a vertebral fracture during Dmab treatment.

### Changes in bone markers during sequential treatment

**Fig. 5:**
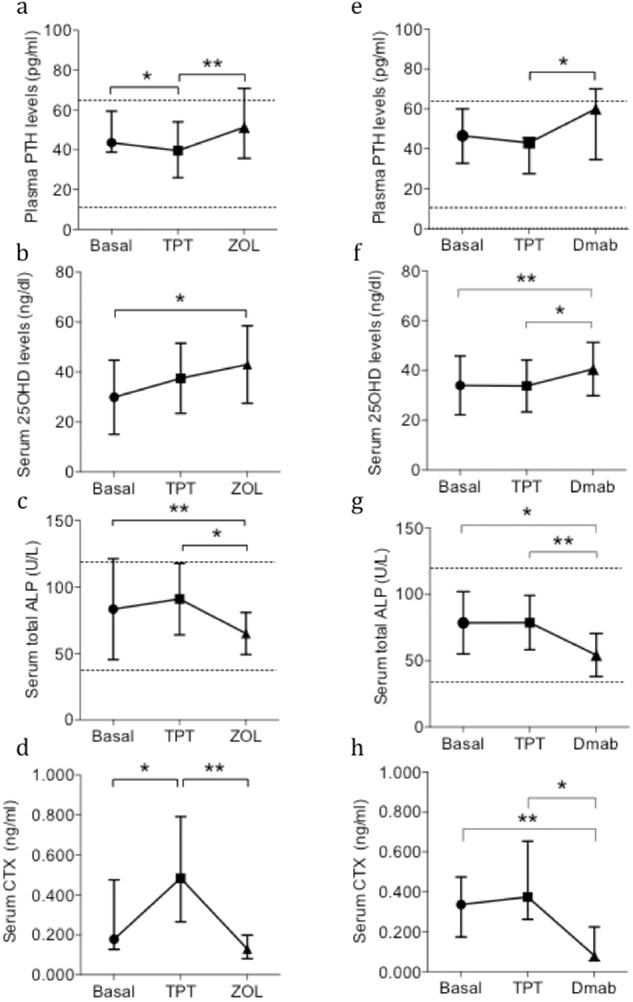
Effects of sequential treatments TPT + ZOL versus TPT+Dmab on circulating bone markers. Plasma PTH levels were reduced by TPT treatment and increased by both ZOL and Dmab treatment (4a, 4e). Serum 25OHD levels were significantly increased during both the sequential treatments (4b, 4f). Serum total ALP and βCTX levels were significantly reduced after TPT + ZOL (4c, 4d) and TPT+Dmab treatments (4g, 4h). Panel (**a**), *p < 0.0001; **p = 0.0007; Panel (**b**), *p = 0.0008; Panel (**c**), *p = 0.0005; Panel (**d**), *p = 0.0133; **p < 0.0001; **p < 0.0001; Panel (**e**), *p = 0.0298; Panel (**f**), *p = 0.0290; **p = 0.0165; Panel (**g**), *p < 0.0001, **p < 0.0001; Panel (**h**), *p < 0.0001; **p = 0.0042. TPT, 20 μg/day sc teriparatide; ZOL, 5 mg/12 months iv zoledronic acid; Dmab, 60 mg/25 weeks sc denosumab

Baseline levels of circulating bone markers were similar between treatment groups (Table 1). Treatment with TPT reduced plasma PTH levels in both treatment groups compared with baseline, although this reduction was only significant in the TPT + ZOL group (Fig. 5a, e). Serum 25OHD levels increased progressively in both treatment groups, likely due to adjustment of the vitamin D supplementation during visits to maintain serum 25OHD levels >30 ng/dl (Fig. 5b, f). As expected, serum total ALP and βCTX levels increased during TPT treatment and decreased during ZOL and Dmab treatment (Fig. 5c, d, g, h). Any significant correlations between circulating bone turnover or mineral markers and the Δ T-scores after TPT + ZOL and TPT+Dmab could be detected.

## Discussion

This retrospective analysis of real-life clinical practice data identified a significant increase in BMD T-scores at the lumbar and hip levels in patients with severe osteoporosis and fragility fractures who received sequential treatment with TPT + ZOL or TPT+Dmab. At the lumbar level, sequential treatment with ZOL conserved the gain in BMD T-score after TPT treatment, effectively preventing the bone loss occurring after TPT discontinuation.

Discontinuation of TPT treatment is known to induce a gradual decline in BMD. Lindsay et al. reported a decrease of almost 3% in lumbar spine BMD at the 1 year-follow-up after discontinuation of TPT in a large study on osteoporotic women treated with TPT 40 μg daily for a mean of 18 months [11]. Similarly, a gradual decline of −1.28% and −1.75% in lumbar spine BMD was observed following discontinuation of TPT 20 μg and 40 μg daily, respectively, after a median of 12 months of treatment in a study of men with osteoporosis; the effect on the total hip BMD was smaller, declining 3% and 2.9% from baseline at month 12, respectively [12]. Both studies highlighted the need for a consolidator treatment to preserve the BMD gain induced by the anabolic agent TPT.

While BPs are known to prevent the decrease in BMD after TPT discontinuation, there is no consensus on which is the best treatment to use, and recommendations in international clinical practice guidelines are lacking [13]. After 12 months of treatment, the oral BP alendronate increased BMD at the lumbar spine by 1.3 ± 5.1% in Japanese men (*n* = 35) and postmenopausal women (*n* = 265) who had received 24 months of treatment with TPT; however, the percent change in lumbar spine BMD was higher with Dmab (4.3 ± 3.5%) [14]. Moreover, BMD continued to increase in postmenopausal osteoporotic women who switched from TPT to Dmab in the DATA-SWITCH study [15], while sequential treatment with Dmab after TPT appeared to yield higher additional lumbar spine BMD gain on average compared with treatment with BPs treatment at 12 months [8].

In our study, the size effect induced by TPT + ZOL on the lumbar BMD T-score was similar to that observed with TPT+Dmab sequential treatment with a mean increase of about 1 SD. Although patients in the TPT+Dmab group had a significantly lower mean lumbar T-score at baseline than patients in the TPT + ZOL group, the change in lumbar T-score induced by sequential treatment did not differ between the two groups. At the femur neck level, neither sequential treatment improved BMD T-scores significantly, while both sequential treatments increased mean T-scores to a similar extent (about 0.4 SD) at the hip level. However, it should be noted that the sequential treatment TPT+Dmab induced T-scores increases in a proportion of patients higher than that observed with the sequential treatment TPT + ZOL.

The data here reported represent the first published study specifically assessing the efficacy of sequential TPT + ZOL and TPT+Dmab in patients with osteoporosis in a real-life setting. Indeed, Sarli et al. [9] previously reported that ZOL and Dmab are effective sequential treatments after TPT, but the study investigated a very limited number of patients treated with a short course of TPT.

It should be noted that, in the present study, patients with low hip T-scores at baseline experienced larger changes in hip T-scores after TPT + ZOL treatment than those with high baseline hip T-scores. Similarly, patients with low neck T-scores at baseline experienced larger neck T-score changes after TPT+Dmab treatment than those with high baseline neck T-scores, demonstrating that both sequential treatments were also effective on bone mineral density at femur site. However, any clinical or circulating bone turnover and mineral marker was predictive of the outcome, in term of T-score changes, of the two sequential treatments.

While the small study size prevents inferences on the fracture incidence, it is notable that both TPT + ZOL and TPT+Dmab sequential treatments prevented further fragility fractures in 87% and 85% of patients, respectively, after 48 months of treatment. This finding is particularly relevant considering that the enrolled patients were elderly and at very high risk of re-fracturing due to the high number of previous fragility fractures and comorbidities.

As expected, analyses of circulating bone markers showed similar patterns of response to both sequential treatments, highlighting the elevated compliance to both sequential treatments registered in the present study.

Admittedly, the study is limited by its retrospective design and the small number of patients. The real-life setting prevented centralized measurements of BMD and of circulating bone and mineral markers, while the use of Δ BMD T-score is less effective in predicting the effectiveness of medications. Nonetheless, the patients were well characterized, both clinically and biochemically, and treated with the same scheduled sequential therapy in the two different arms. It is also of interest that the efficacy of both sequential treatments was demonstrated in patients with severe fractured osteoporosis at very high risk of fragility re-fractures.

## Conclusions

Sequential therapy with TPT + ZOL is likely to be effective in increasing bone mineralization at the lumbar and hip levels. This effect of TPT + ZOL is not inferior to that observed with sequential TPT+Dmab treatment, suggesting that TPT + ZOL sequential treatment is a valid alternative therapeutic option to treat osteoporosis, which, at variance with TPT+Dmab treatment, does not require further consolidation if ZOL is discontinued.

## Data Availability

The data that support the findings of this study are openly available in “zenodo” at 10.5281/zenodo.7248449.

## Abbreviations:

25OHD: 25 hydroxyvitamin D
ALP: alkaline phosphatase
BMD: bone mineral density
BPs: bisphosphonates
βCTX: carboxy-terminal cross-linked telopeptide of type I collagen
Dmab: denosumab
DXA: dual-energy x-ray absorptiometry
IR: interquartile range
PTH: parathormone
SD: standard deviation
TPT: teriparatide
ZOL: zoledronic acid
AIFA: Agenzia Italiana del Farmaco.
